# Skin layer thickness, muscle elasticity and their effects on pain level in fibromyalgia patients

**DOI:** 10.1016/j.clinsp.2025.100858

**Published:** 2025-12-30

**Authors:** Selçuk Akan, Mehtap Balaban, Ahmet Kor, Bahadir Erturk

**Affiliations:** a Ankara City Hospital, Physical Medicine and Rehabilitation Hospital, İnternal Medicine Department, Ankara, Turkey; b Yıldırım Beyazıt Universıty, Ankara City Hospital, Physical Medicine and Rehabilitation Hospital, Radiology Department, Ankara, Turkey; c Aksaray Training and Research Hospital, Rheumatology Department, Aksaray, Turkey; d Kaman District Health Directorate, Family Medicine, Kırşehir, Turkey

**Keywords:** Fibromyalgia, Ultrasonography, Visual analogue scale, Skin thickness, Muscle thickness, Sonoelastography

## Abstract

**Background:**

Fibromyalgia (FM) is a chronic, non-inflammatory syndrome characterized by widespread body pain, fatigue, sleep disturbances, impaired cognitive function and anxiety. Peripheral and central sensitization are thought to cause chronic pain in this disorder, which impairs quality of life. No specific laboratory test, radiographic method, or biomarker has been identified for diagnosis. As its physiopathology is not fully understood, no specific treatment has been identified. At this stage, studies are needed to facilitate diagnosis and guide treatment.

**Methods:**

Eighty-nine women with FM and 36 healthy controls were enrolled in this cross-sectional, case-controlled, single-centre study. FM-related measurements (number of tender points, myalgic score, pain Visual Analogue Scale [VAS]) were recorded. All participants underwent an analysis of trapezius muscle thickness and elasticity. Skin and subcutaneous tissue thickness, and hypodermis thickness were measured by using B-mode Ultrasonography (USG) and tension (compression) Sonoelastography (SEL).

**Results:**

Skin thickness was lower in FM patients than in healthy controls (*p* < 0.01). Both trapezius muscle thickness and elastography scores were higher in the FM group compared to controls (*p* < 0.05 and *p* < 0.001, respectively). A significant correlation was found between VAS score and elastography scores (*r* = 0.661, *p* < 0.001); however, there was no significant correlation between VAS score and trapezius or skin thickness.

**Conclusion:**

Patients with FM have lower skin thickness and higher trapezius muscle thickness and elastography scores than healthy controls. The thinner skin thickness in FM compared to controls may represent a potentially important factor in the pathophysiological mechanism of peripheral sensitization.

## Highlights

• Fibromyalgia patients have thinner skin on ultrasound.
• Trapezius muscle is thicker and stiffer in fibromyalgia.
• Muscle stiffness is correlated with pain severity.
• Reduced skin thickness may drive peripheral sensitization.

## Introductıon

Fibromyalgia (FM) is a chronic (>3-months), non-inflammatory, non-autoimmune disorder of peripheral and central afferent processing that causes a widespread pain syndrome. Core symptoms typically include widespread pain, fatigue, stiffness, sleep disturbances, cognitive problems (fibrofog), and psychological impairments (depression, anxiety, or both). Physical and pathological examinations show no evidence of inflammation or degeneration of joints, bones, or soft tissues. Patients may have tender points upon examination, but these are no longer included in the diagnostic criteria as they lack sensitivity and specificity for FM.^1^

FM can occur alone (primary FM) or be associated with other conditions (secondary FM). In primary FM, laboratory tests and radiographs are normal.^2^ In this debilitating disease, peripheral and central sensitization are believed to cause chronic pain. It is thought that peripheral polymodal C-fibers and silent nociceptive neurons, previously unresponsive to stimulation, become responsive and that nociceptors begin to produce spontaneous signals. Thus, due to the lowered pain threshold (peripheral sensitization), thermal, mechanical, or chemical stimuli now cause diffuse pain specific to FM.^3^

Although many studies have investigated the relationship between FM and muscles, the results are inconclusive. Studies designed to elucidate the pathophysiology of chronic muscle pain, muscle stiffness, and muscle fatigue ‒ main outcomes of Fibromyalgia Syndrome (FMS) ‒ have produced mixed findings. The deltoid, trapezius, brachioradialis, tibialis anterior, and quadriceps muscles have been evaluated using direct methods (e.g., histochemical, electrophysiological, magnetic resonance imaging) and indirect methods (e.g., functional assessment, muscle strength measurements). Some studies reported mild pathophysiological findings such as mitochondrial abnormalities, capillary insufficiency, microcirculatory disturbances, and muscle fiber changes^4^, ^5^, ^6^, ^7^, ^8^, ^9^, ^10^, ^11^, ^12^ while others found no significant differences between FM patients and healthy controls.^6^^,^^13^^,^^14^

One study showed that muscles with tender points were affected,^15^ while two others reported that asymptomatic muscles were not affected.^16^^,^^17^

B-mode ultrasound and ultrasound elasography are affordable, non-radiating, reliable diagnostic tools that can be easily and quickly performed. These tools can serve as biomarkers to determine skin and subcutaneous tissue thickness, muscle thickness, and muscle elasticity (stiffness).^18^^,^^19^

Elasticity refers to the degree to which an externally applied force can change the original shape and size of a tissue. In ultrasound elastography, ultrasound-focused mechanical vibrations are transmitted into the tissue, and the velocity of waves generated in tissues of different elasticity is measured. A color-coded qualitative map is produced.^20^, ^21^, ^22^

To date, muscle changes associated with chronic pain in FM have been evaluated using histological, electrophysiological, and imaging methods, with conflicting results regarding the role of muscles in FM.^23^ While studies on the elastographic evaluation of muscles in FM are limited, ultrasonographic evaluation of the skin layers has not yet been performed. Given that the peripheral nerve network ‒ central to the peripheral sensitization mechanism in FM^3^ ‒ runs through the epidermal and dermal layers,^24^ skin layer thickness may play a role in FM. In this study, the authors evaluated and compared the skin-subcutaneous thickness, muscle thickness, and muscle elasticity of FM patients and healthy controls using B-mode US and US elastography. The authors also investigated the potential role of skin thickness and muscle elasticity in the pathophysiology of FM and their correlation with the VAS pain score.

## Materıals and methods

### Participants

This was a cross-sectional, case-controlled, single-center study conducted at Ankara City Hospital. Data were collected between January 2022 and January 2023. The study included 89 female patients who were newly diagnosed with FMS according to the 2016 American College of Rheumatology (ACR) diagnostic criteria,^25^ who presented to the departments of internal medicine, physical medicine and rehabilitation, and the rheumatology outpatient clinic of the hospital. Thirty-six healthy women were also included as a control group. All subjects were between 18- and 55-years old and volunteered to participate in the study. Exclusion criteria included spinal pain due to cervical stenosis, spinal root or cord compression, a history of neurological or inflammatory rheumatic disease, pregnancy, cognitive or communication problems, history of physical therapy and rehabilitation in the last 3-months, or use of agents that decrease or increase muscle tone (e.g., muscle relaxants, more than one cup of coffee, alcohol, drugs, etc.) within 12-hours prior to measurements. Before the study, participants were informed about the study and their written consent was obtained. The study was approved by the hospital’s ethics committee (date: 19.01.2022; number: E2–22–1281). Demographic and physical characteristics (age, BMI, duration of symptoms, sex, education, employment status, smoking status and painful areas) were recorded. This cross-sectional study was conducted and reported in accordance with the STROBE Statement guidelines.

### Pain evaluation

Pain intensity was assessed using the VAS by a clinician. Pain intensity at rest, during activity and at night was evaluated on a Visual Analogue Scale (VAS). Participants were asked to place a mark on a 10 cm straight line according to the intensity of pain they experienced. These marks were measured from the starting point using a ruler and recorded in “centimeters” (cm). A score of 0 cm indicated ‘no pain’, 1–3.9 cm indicated ‘mild pain’, 4–6.9 cm ‘moderate pain’, 7–10 cm ‘severe pain’, and 10 cm ‘unbearable pain’. Patients’ VAS pain intensities were determined and recorded. Ultrasonographic analyses were performed by an experienced musculoskeletal radiologist to assess trapezius muscle thickness, muscle elasticity, and skin and subcutaneous tissue thickness.

### Ultrasonographic measurements

**Fig. 1:**
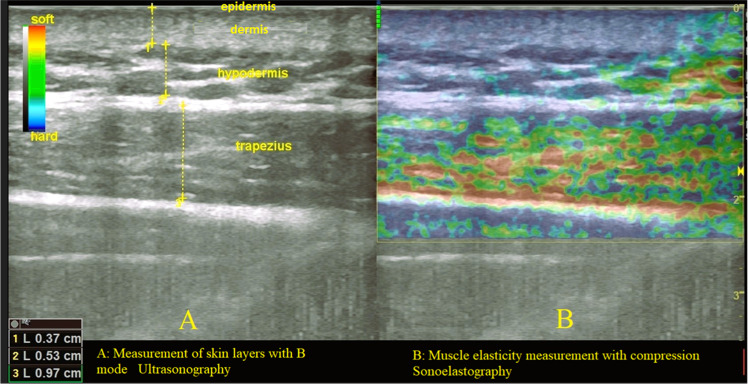
Measurement of skin-subcutaneous tissue thickness with B mode Ultrasonography and muscle elasticity measurement with Sonoelastography.

Trapezius (TRP) muscle stiffness, skin-subcutaneous tissue thickness, and subcutaneous adipose tissue anterior to the muscle were assessed using B-mode Ultrasonography (US) and tensile (compression) Sonoelastography (SEL) by an experienced radiologist with at least 15-years' experience using a Logiq S7/Expert (General Electric, Toronto, Canada) ultrasound machine. Measurements were performed in real time using an 11 MHz linear transducer probe. Care was taken not to apply excessive pressure. For evaluation, the probe was placed 2 cm lateral to the midpoint of the acromion of the scapula and the spinous process of the 7th cervical vertebra with the subjects in the prone position. Skin-subcutaneous tissue and subcutaneous fat thicknesses were measured by measuring the anteroposterior diameter using B-mode US. To reduce measurement error, muscle thickness, skin-subcutaneous tissue thickness and subcutaneous tissue thickness measurements were repeated 3-times and the average of the 3 measurements was taken. The results of the SEL measurements were simultaneously displayed on a color map superimposed on the B-mode image (Fig. 1). Measurement results were evaluated using a 5-point color classification scale described in the literature. The measurements were graded according to this scale, with “1″ being the softest and “5″ being the hardest. The area represented in a color map superimposed on the B-mode image was defined as “1″ (soft) when red color was dominant, “2″ (mostly soft) when red color was dominant and green color was present up to 1/4, “3″ (medium hardness) when green color was dominant, “4″(mostly hard) when blue color was dominant and green color was present up to 1/4, and “5” (hard) when blue color was dominant. Measurement results were recorded according to the side with greater pain intensity (right or left TRP) based on the 5-point color classification scale. For individuals with equal pain on both sides, the dominant side was used for analysis.

1 (A) Most elastic (soft): Predominantly red.
2 (B) More elastic (mostly soft): Green with up to 25 % red.
3 (C) Intermediate elasticity: Predominantly green.
4 (D) Less elastic (mostly hard): Green with up to 25 % blue.
5 (E) Least elastic (hard): Predominantly blue.

### Statistics

In the study, continuous variables were presented as means and standard deviations, while categorical variables were presented as frequencies and percentages. The distribution of data was assessed using the Kolmogorov-Smirnov test and box plots. For pairwise comparisons, continuous variables were evaluated using the Student t-test, and categorical variables were evaluated using the chi-square test. Correlations between symptom severity and US measurements were assessed by Pearson correlation analysis. For all analyses, *p* < 0.05 was accepted as the statistical threshold.

## Results

All individuals completed the study. There were no statistically significant differences between the two groups in terms of age, Body Mass Index (BMI) and smoking habits (*p* = 0.341, *p* = 0.810, *p* = 0.091). The mean VAS pain score of fibromyalgia patients was above 7. Right and left arm use was similar in patients and healthy controls. All laboratory parameters measured in the patient and control groups were within normal reference ranges. Demographic distribution and analysis of laboratory parameters are shown in Table 1.

**Table 1:** Comparison of demographic distribution and laboratory parameters between groups.

**Parameter**		**Group**		**p**
		**HC (*n* = 36)**	**FP (*n* = 89)**	
Age (year)		44.36 ± 9.96	42.46 ± 10.5	0.341
BMI (kg/m²)		27.46 ± 4.76	27.23 ± 5.06	0.810
To smoke n ( %)	Yes	13 (37.14 %)	27 (30.34 %)	0.091
	No	22 (62.86 %)	62 (69.66 %)	
Dominant used arm n ( %)	Right	15 (41.7 %)	36 (40.5 %)	0.473
	Left	12 ( %33.3)	29 (32.6 %)	
	Right and Left	9 (25 %)	24 (26.9 %)	
VAS (0‒10) (Mean ± SD)		0	7.43 (1.89)	
Hemoglobin (mg/dL) (Mean ± SD)		12.56 ± 1.41	13.11 ± 1.43	**0.038**
Glucose (mg/dL) (Mean ± SD)		95.46 ± 32.59	97.22 ± 36.20	0.779
Creatinine (mg/dL) (Mean ± SD)		0.64 ± 0.10	0.71 ± 0.10	**0.001**
Albumin (g/dL) (Mean ± SD)		45.15 ± 2.43	46.19 ± 5.27	0.123
CPK (U/L) (Mean ± SD)		84.55 ± 59.95	69.58 ± 36.10	0.179
AST (U/L) (Mean ± SD)		22.11 ± 6.96	23.18 ± 11.91	0.515
ALT (U/L) (Mean ± SD)		22.65 ± 11.03	23.27 ± 13.97	0.783
Calcium (mg/dL) (Mean ± SD)		9.56 ± 0.40	9.50 ± 0.42	0.475
Magnesium (mg/dL) (Mean ± SD)		1.92 ± 0.18	2.01 ± 0.35	0.058
TSH (µIU/L) (Mean ± SD)		2.51 ± 3.61	1.84 ± 1.13	0.255
Vitamin D (ng/mL) (Mean ± SD)		47.66 ± 26.36	47.59 ± 24.52	0.988
Vitamin B12 (ng/mL) (Mean ± SD)		390.79 ± 609.25	330.45 ± 74.64	0.545

ALT, Alanine Aminotransferase; AST, Aspartate Aminotransferase; BMI, Body Mass Index; CPK; Creatine Phospo Kinase; FP, Fibromyalgia Patients; HC, Health Control Group; SD, Standard Deviation; TSH, Thyroid Stimulating Hormone; VAS, Visual Analogue Scale.

Table 2 compares trapezius muscle thickness, trapezius elasticity, skin-subcutaneous tissue thickness and hypodermis thickness on the dominant side in FM patients and healthy controls. Trapezius muscle thickness was statistically significantly higher in fibromyalgia patients (*p* = 0.035), while skin-subcutaneous tissue thickness was higher in the healthy group (*p* = 0.006). There was no difference in hypodermis thickness between the groups (*p* = 0.106). In terms of trapezius elasticity, fibromyalgia patients had a higher score of 3-points in terms of trapezius elasticity, while 2-points were observed more frequently in the control group (*p* < 0.001). While 2 patients in the healthy group scored 1-point, 1-patient in the FM group scored 1-point, 2-patients scored 4-points and 2-patients scored 5-points. These results showed that the trapezius muscle thickness, skin-subcutaneous tissue thickness and trapezius elasticity of FM patients were significantly different from those of non-patients.

**Table 2:** Comparison of ultrasonographic measurements of skin and muscles in fibromyalgia patients and healthy controls.

Parametre	Elasticity level	Group		p
		HC (*n* = 36)	FP (*n* = 89)	
Skin-Subcutaneous Thickness, mm (Mean ± SD)		2.58 ± 0.67	2.28 ± 0.46	**0.006**
Hypodermis Thickness, mm (Mean ± SD)		7.16 ± 2.30	6.46 ± 2.15	0.106
Trapezius Thickness, mm (Mean ± SD)		6.93 ± 1.20	7.49 ± 1.39	**0.035**
Trapezius Elasticity, n ( %)	1	2 (5.6 %)	1(1.1 %)	
	2	**31 (86.1%)**	**31 (34.9%)**	**<0.001**
	3	**3 (8.3%)**	**53 (59.6%)**	
	4	0	2 (2.2 %)	
	5	0	2 (2.2 %)	

FP, Fibromyalgia Patients; HC, Health Control Group; SD, Standard Deviation.

Table 3 shows the correlations between ultrasound findings and trapezius elasticity scores and VAS scale. While there was a significant correlation between trapezius elasticity scores and VAS scale (*p* < 0.001), there was no significant correlation between ultrasound parameters and elasticity scores and VAS scale (*p* > 0.05 for each).

**Table 3:** Correlations between elasticity and VAS pain scale and ultrasonographic measurements in fibromyalgia patients.

		Trapezius Thickness	Skin-subcutaneous thickness	Hypodermis Thickness	Trapezius Elasticity Score
**Elasticity Score**	*r*	0.127	0.138	0.116	
	p	0.236	0.199	0.280	
**VAS**	*r*	0.123	0.183	0.041	0.661
	p	0.253	0.085	0.709	**<0.001**

VAS, Visual Analogue Scale.

## Discussion

This study demonstrated that patients with FM have significantly lower skin thickness and significantly higher trapezius muscle thickness and muscle stiffness (elastography scores) than healthy controls. In addition, a significant correlation was found between trapezius elasticity scores and the VAS scale (*p* < 0.001), whereas no correlation was found between ultrasound parameters and elasticity scores and the VAS scale (*p* > 0.05 for each).

Although patients with FM and healthy subjects have not previously been compared in terms of skin thickness, studies have shown that pathological changes occur in peripheral nervous system structures in the epidermal/dermal region. These studies have reported a significant decrease in intraepidermal nerve fibre density in a large percentage of patients,^26^^,^^27^ reflecting the loss of the most distal nociceptors.^28^ It has been suggested that this pathogenic condition may explain some clinical findings associated with peripheral autonomic dysfunction.^29^^,^^30^

The mechanism of abnormalities in peripheral nociceptors in FM and their implications in clinical practice remain an unsolved problem.^26^ In the study, the epidermis/dermis layers were found to be thinner in the FM patient groups compared with controls. The question of whether the thinning of the skin in FM is caused by abnormalities in the peripheral nerve structures or, conversely, whether abnormalities in the skin layers cause damage to the intra-epidermal/dermal nerve networks is an interesting area of research. Further research into the pathological interactions between skin layers and peripheral nervous system structures in FM will provide clarifying answers to these questions.

The present study found that trapezius muscle thickness was higher in patients with FM compared to healthy controls. However, no significant correlation was found between trapezius muscle thickness and VAS score. There is a limited number of studies in the literature that have used ultrasound to assess muscle thickness in patients with FM. In the study by Kuzu O. et al., cervical extensor muscle groups and trapezius muscle thicknesses were evaluated, and muscle thicknesses were found to be lower in FM patients compared to healthy controls. Similar to the present results, no significant correlation was found between disease indices and trapezius muscle thickness.^31^ In the study by Umay E et al., upper and lower extremity muscle thicknesses, excluding the trapezius, were compared between FM and healthy groups; deltoid muscle thickness was similar between groups, whereas other muscle thicknesses were found to be lower in FM patients. This study found a significant correlation between disease indices and quadriceps femoris and biceps brachii thicknesses, whereas no correlation was found between other muscle groups and disease indices.^24^

The literature data show inconsistent results between muscle thickness and disease indices in FM. Similarly, studies of muscle strength in FM patients have shown conflicting results, with some studies reporting loss of muscle strength^32^^,^^33^ and others reporting normal muscle strength.^34^^,^^35^ Different results may be due to insufficient numbers of patients, different disease durations and dominant extremities, and the presence of other factors (age, ethnicity, occupational and social status, etc.).

In the study, muscle thickness was found to be higher in fibromyalgia patients than in controls, in contrast to previous studies. The results may have been different because our study included newly diagnosed patients and measured the dominant limb muscles. This is because muscle thickness changes depending on how the muscles are used, and thickness is greater on the dominant side. In addition, it has been reported that muscle wasting occurs in this patient group as a result of immobilization due to pain and decreased physical performance with increasing disease duration.^36^^,^^37^ However, the clinical or pathological effects of ultrasound muscle thickness in FM need to be investigated in further studies.

In our study, Fibromyalgia (FM) patients had significantly higher hemoglobin and creatinine levels compared to healthy controls. Although both values remained within normal reference ranges, this finding may reflect subtle alterations in muscle metabolism and renal function. Previous studies indicate that increased muscle catabolism or reduced renal efficiency may underlie minor elevations in serum creatinine even without overt kidney disease.^38^

Similarly, elevated hemoglobin could arise from hemoconcentration due to chronic pain-related stress, reduced plasma volume, or compensatory autonomic responses. Fibromyalgia has been linked with autonomic nervous system dysfunction, sympathetic hyperactivity, and vascular dysregulation, which may induce hemoconcentration and higher hemoglobin values in some patients.^39^ While these laboratory findings alone are not diagnostic, they may provide additional insights into the systemic manifestations of fibromyalgia and should be further explored in future studies.

Elastography is an accepted method for assessing static or dynamic muscle function. According to the results, trapezius muscle elastography scores were higher in patients with FM than in controls. There was also a significant positive correlation between elastography scores and VAS. One elastography study reported that stiffness during muscle contraction increased in young people and did not change in older people.^40^ Elastography studies in FM patients are limited. Muro-Culebras and Cuesta-Vargas used ultrasound and elastography to assess the morphology, stiffness and blood flow of tender points in 16-women with FM. They found no significant difference in the number of elliptical and hypoechoic areas, blood flow, or stiffness in the trapezius muscle of FM patients compared with controls.^41^ In another study, the rhomboid muscles of 53 patients with FM were assessed by elastography, and similar to our results, elastography scores were found to be higher in patients with FM compared to healthy controls. In addition, no significant correlation was found between VAS and elastography scores in this study.^23^ It has been reported that muscles are often affected in chronic pain conditions such as FM, and these patients have higher elastographic scores compared to healthy individuals due to increased muscle spasticity.^23^^,^^42^ Although the literature data give conflicting results regarding the elastographic scores of the muscular structures of patients with FM, the clinical use and usefulness of elastography in the FM population needs to be clarified. Further studies should therefore be undertaken.

In order to provide a broader perspective on the muscular involvement in fibromyalgia and to contextualize our results, Table 4 summarizes selected studies that have evaluated muscle structure, thickness, or elasticity in FM patients.^4^^,^^5^^,^^22^^,^^23^^,^^31^^,^^41^^,^^43^^,^^44^ These studies vary in terms of muscle groups evaluated, imaging methods applied, and reported outcomes. While some report reduced muscle thickness or no significant differences, others ‒ especially those using elastography ‒ have demonstrated increased muscle stiffness. Our study aligns with the latter, showing increased trapezius stiffness and a positive correlation with pain intensity.

**Table 4:** Summary of selected studies evaluating muscles in fibromyalgia patients.

Study	Muscle(s) Evaluated	FM / Control (n)	Method	Main Findings
Meral et al. 2025	Trapezius	34 / 34	B-mode US. echotexture analysis	Increased echogenicity and blob size in FM; correlated with VAS and CSI
Mesci et al. 2023	Gastrocnemius trapezius. upper arm	22 / 18	B-mode US	Reduced muscle thickness in FM; associated with functional measures
Kuzu et al. 2022	Multifidus. semispinalis capitis. semispinalis cervicis. splenius capitis and trapezius muscles	41/41	B-mode US	Lower trapezius thickness in FM; no correlation with disease indices
Karayol et al. 2021	Rhomboid	53 / 47	Shear wave elastography	Cervical extensor muscle thickness in patients with FM; no correlation with VAS
Umay et al. 2020	Quadriceps. biceps brachii. deltoid	102 /50	B-mode US	Decreased thickness in quadriceps and biceps in FM; deltoid unchanged
Muro-Culebras et al. 2013	Trapezius	16 / 16	Sono-myography. sono-myoelastography	No significant difference in stiffness or blood flow between groups
Srikuea et al. 2013	Quadriceps	11/11	Histochemistry. biopsy	Mitochondrial abnormalities and inflammatory infiltrates in FM muscle tissue
Klaver-Kröl et al. 2012	Biceps brachii	13/13	Electrophysiological tests	Altered muscle membrane function in FM associated with tender point count
Current study (2025)	Trapezius. skin layers	89 / 36	B-mode US + SEL elastography	Increased trapezius thickness and stiffness. decreased skin thickness; SEL correlated with VAS

FM, Fibromiyalgia; US, Ultrasonography; SEL, Sonoelastography; VAS, Visual Analogue Scale.

This study has several limitations. The cross-sectional design precludes any conclusions about causality, and the elastographic findings were not corroborated by MRI or histopathological analysis. Furthermore, although an experienced radiologist performed the ultrasonographic measurements, blinding to the patient fibromyalgia status was not implemented, potentially introducing observer bias. However, standardized measurement protocols and averaging multiple measurements were employed to mitigate this risk. Despite these limitations, this study provides novel evidence that skin thickness is reduced in FM patients compared to healthy controls, and it supports previous findings of elevated elastographic muscle scores in FM.

## Conclusion

Patients with FM have lower skin thickness and higher trapezius muscle thickness than healthy controls. In addition, trapezius muscle elastography scores were higher in patients than in controls and correlated with VAS. This study highlighted that the thinner skin thickness in FM compared to controls may represent a possible important step in the pathophysiological process involved in the mechanism of peripheral sensitization.

## Data sharing statement

The datasets generated and analyzed during the current study are available from the corresponding author on reasonable request. All relevant data are included in this published article.

## Funding

The authors declared that this study received no financial support.

## Data availability

The datasets generated and/or analyzed during the current study are available from the corresponding author upon reasonable request.

## CRediT authorship contribution statement

**Selçuk Akan:** Conceptualization, Methodology, Software, Formal analysis, Writing – original draft, Writing – review & editing, Supervision. **Mehtap Balaban:** Methodology, Investigation, Data curation, Visualization. **Ahmet Kor:** Investigation, Writing – review & editing. **Bahadir Erturk:** Software, Validation.

## Declaration of competing interest

The authors declare no conflicts of interest.
